# Generic nutritional risk index and urinary incontinence in U.S. older adults: A cross-sectional analysis of NHANES 2001–2018

**DOI:** 10.1097/MD.0000000000050584

**Published:** 2026-09-04

**Authors:** Juan Xu, Shangqi Cao, Xu Hu, Xiang Li, Shu Wen

**Affiliations:** a Operating Room, West China Hospital, Sichuan University/West China School of Nursing, Sichuan University, Chengdu, Sichuan, China; b Department of Urology, Institute of Urology, West China Hospital, Sichuan University, Chengdu, Sichuan, China; c Department of Critical Care Medicine, West China Hospital, Sichuan University, Chengdu, Sichuan, China.

**Keywords:** geriatric nutritional risk index, National Health and Nutrition Examination Survey, NHANES, nutrition, urinary incontinence

## Abstract

This study aims to evaluate the association between the geriatric nutritional risk index (GNRI) and urinary incontinence (UI). This cross-sectional study utilized data derived from 9 cycles (2001–2018) of the National Health and Nutrition Examination Survey. Weighted multivariable logistic regression models were employed to assess the connection between GNRI and UI [stress UI (SUI), urgency UI (UUI), and mixed UI (MUI)]. Smooth curve fitting was conducted to explore the linear association between GNRI and UI. Subgroup analysis was used to assess the robustness of the relationship between GNRI and UI across various stratifications. In the current study, a total of 11,324 participants older than 60 years were enrolled, with 32.2% experiencing SUI, 36.5% complaining of UUI, and 17.8% having MUI. In the multivariable logistic regression, GNRI was negatively associated with 3 types of UI with full adjustment (SUI: odds ratio [OR] = 0.986, 95% confidence interval [CI]: 0.974–0.999; UUI: OR = 0.972, 95% CI: 0.961–0.983; MUI: OR = 0.972, 95% CI: 0.957–0.987, all *P* < .05). After categorizing GNRI into quartiles, a significant negative relationship between GNRI and UI was stable, particularly evident in the highest GNRI group when compared to the lowest GNRI quartile (SUI: OR = 0.797, 95% CI: 0.672–0.945; UUI: OR = 0.722, 95% CI: 0.615–0.848; MUI: OR = 0.674, 95% CI: 0.538–0.845, all *P* for trend < .05). Additionally, the smooth curves fitting revealed a negative linear connection between GNRI and UI. Moreover, the negative association between GNRI and SUI, as well as MUI, was more significant in men than in women (*P* for interaction < .05). In conclusion, GNRI was inversely associated with the prevalence of UI. The association between GNRI and SUI, as well as MUI, was stronger in males compared to females. Further research is needed to confirm causality and explore potential mechanisms.

## 1. Introduction

Urinary incontinence (UI) is the involuntary leakage of urine, which is common in older adults but can also affect younger adults and significantly impacts both health and quality of life.^[1]^ Approximately 24 to 45% of women reported UI; in women older than 60, approximately 9 to 39% reported UI on a daily basis.^[1]^ And UI has a reported prevalence of 11% in men aged 60 to 64 years and 31% in men ≥ 85 years of age.^[2]^ UI can be classified into stress UI (SUI), urgency UI (UUI), and mixed UI (MUI). SUI indicates the involuntary discharge of urine during physical exertion, coughing, or sneezing.^[3]^ UUI refers to the involuntary voiding of urine accompanied by a sudden urge. MUI constitutes a condition wherein involuntary urine leakage occurs with urgency, as well as with physical activities, coughing, or sneezing.^[4]^ Various risk factors contribute to UI, encompassing but not confined to aging, complications arising from childbirth, medications, sedentary lifestyle, and obesity.^[5]^ Although UI significantly impacts quality of life, its underlying causes and mechanisms are not yet fully understood.

An increasing number of studies have shown an association between UI and nutrient intake. A cross-sectional study conducted by Yuan et al demonstrated that the excessive intake of added sugar among women may increase their risk of SUI and MUI.^[6]^ A propensity scores matching study involving 8090 women showed that UI was associated with high carbohydrate intake.^[7]^ They think it may be associated with obesity and blood sugar fluctuations brought on by carbohydrates. Choi et al revealed that unhealthy dietary habits (such as a preference for salty foods, frequent intake of sweets, and insufficient vegetable consumption) are associated with the occurrence and severity of lower urinary tract symptoms.^[8]^ Xu et al found that women with higher serum albumin levels had a lower risk of SUI, suggesting that higher nutritional status may reduce the risk of developing SUI.^[9]^ Nutrient levels seem to play a role in the occurrence of UI.

The geriatric nutritional risk index (GNRI), a simple and objective method for assessing the nutritional status of older adults, can be easily calculated using serum albumin levels and ideal body weight.^[10]^ Studies have found that GNRI is a better predictor of nutritional status than body mass index (BMI) and serum albumin level alone.^[11]^ Multiple studies have found that GNRI has a significant predictive role in the prognosis of cardiovascular disease and tumors.^[12–17]^ GNRI, as an indicator that better reflects nutritional status, reduces the mutual confounding of BMI and serum albumin. However, to our knowledge, the relationship between GNRI and UI has not been explored. This study will focus on the association using data from the National Health and Nutrition Examination Survey (NHANES) database.

## 2. Materials and methods

### 2.1. Study description and population

The data utilized in this study were obtained from the NHANES dataset, a prominent population-based initiative conducted by the Centers for Disease Control and Prevention’s National Center for Health Statistics. The primary objective of NHANES is to assess the health and nutritional status of the United States (U.S.) population, and a distinctive feature of its survey is the combination of interviews and physical examinations. The NHANES employed a sophisticated stratified multistage probability design to gather a nationally representative sample from the noninstitutionalized civilian population of the U.S. More detailed information can be accessed at: https://www.cdc.gov/nchs/nhanes/index.html.

**Figure 1.**
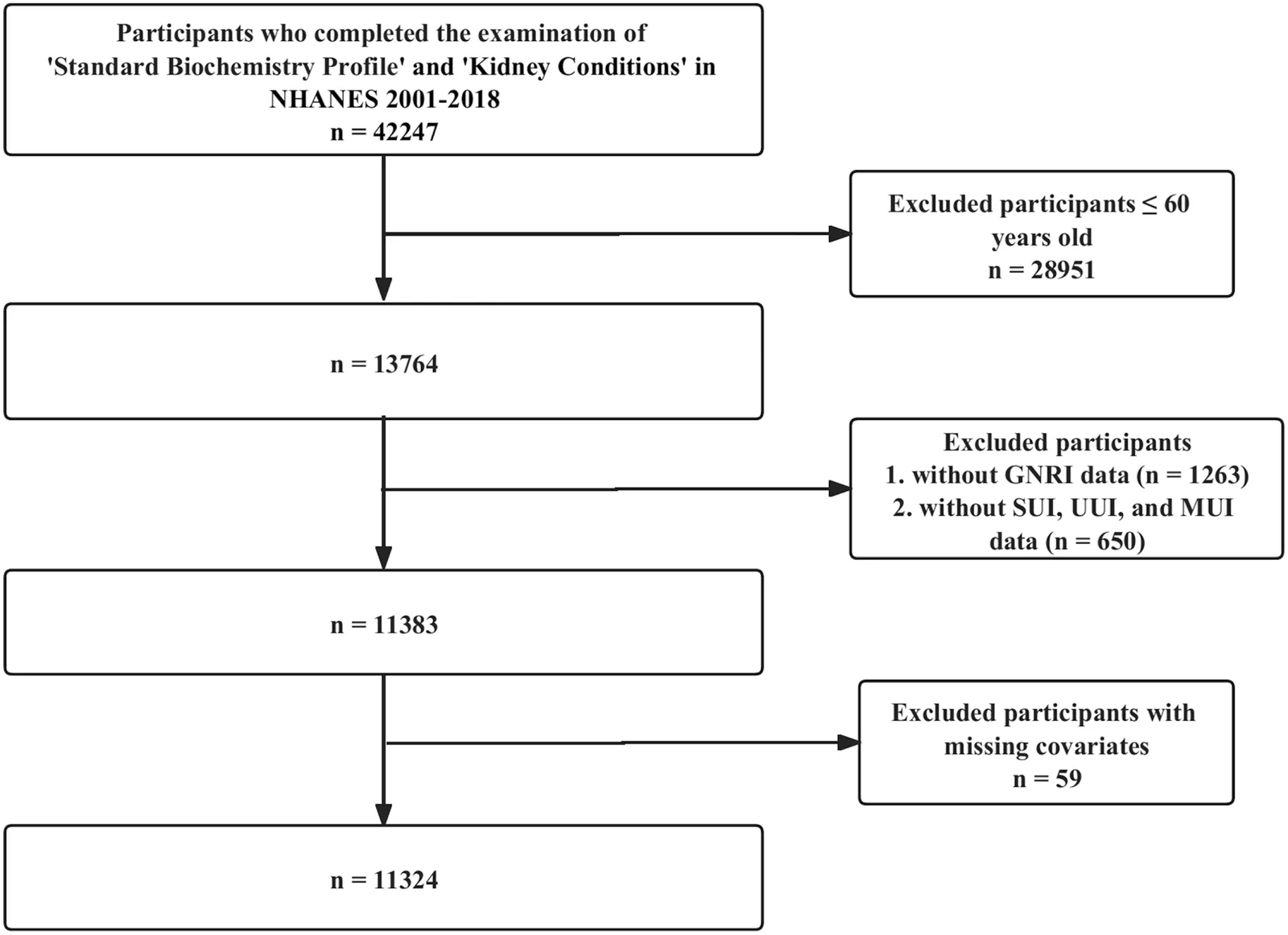
Flow chart of sample selection process. GNRI = geriatric nutritional risk index, MUI = mixed urinary incontinence, n = number of participants, NHANES = National Health and Nutrition Examination Survey, SUI = stress urinary incontinence, UUI = urgency urinary incontinence.

In this study, we obtained data from 9 NHANES cycles (2001–2018) and included a total of 42,247 participants at first. Survey individuals aged ≤ 60 years old (n = 28,951), without GNRI data (n = 1,263), without UI information (n = 650), and with missing covariates (n = 59) were excluded. Finally, 11,324 participants were enrolled in this study (Fig. 1).

### 2.2. Measurement of GNRI

GNRI is a risk index related to nutritional status and serves as a strong prognostic indicator for morbidity and mortality, especially in hospitalized older adults. GNRI was calculated as: GNRI = [1.489 × albumin (g/L)] + {41.7 × [present weight (kg)/ ideal weight (kg)]}. The ideal weight was defined as height^2^ (m^2^) × 22 (kg/m^2^).^[10]^ Generally, a higher GNRI meant the higher nutrition-related risk. Based on the quartile of GNRI, GNRI was divided into 4 groups (Q1, Q2, Q3, Q4). GNRI was set as a continuous and categorical variable in the analyses.

### 2.3. Assessment of UI

The status of UI was evaluated by 2 questions in the “Kidney Condition” in the NHANES. Survey individuals who answered “yes” to the question “During the past 12 months, have you leaked or lost control of even a small amount of urine with an activity like coughing, lifting, or exercise?” were defined as having SUI. Participants were set as having UUI when they said “yes” to the question “During the past 12 months, have you leaked or lost control of even a small amount of urine with an urge or pressure to urinate and you couldn’t get to the toilet fast enough?.” Furthermore, if participants answered “yes” to both questions, they were regarded as having MUI.

### 2.4. Covariates of interest

In this study, gender, age, race/ethnicity, education level, marital status, the family poverty-to-income ratio (PIR), BMI, smoking status, alcohol intake, vigorous activity, moderate activity, diabetes, and hypertension were designed as covariates of interest. In order to avoid the reduction of the substantial sample size within our study, we treated numerous missing covariates, the family PIR (n = 1024) and alcohol intake (n = 1714), as missing value categories, which were subsequently encoded as dummy variables in our regression models. Participants meeting the criterion of having smoked at least 100 cigarettes throughout their lifetime and presently smoking every day or some days at the time of the interview were categorized as current smokers. Those who acknowledged a history of having smoked at least 100 cigarettes but were not currently smoking at the time of the questionnaire were designated as former smokers. Furthermore, for the classification of survey respondents as nonsmokers, it was stipulated that individuals reporting smoking fewer than 100 cigarettes over their lifetime would be encompassed within this category. Participants were stratified into 2 distinct groups: the “drinkers,” defined as individuals who reported an annual consumption of a minimum of 12 alcoholic beverages, and the “nondrinkers.” Survey participants who had received a medical diagnosis of diabetes prior to the interview or exhibited a fasting plasma glucose level ≥ 126 mg/dL were categorized as individuals with diabetes. Participants were classified as having hypertension if they had received a medical diagnosis from doctors, were under medication for hypertension, or presented with a systolic blood pressure level ≥ 140 mm Hg or a diastolic blood pressure level ≥ 90 mm Hg.

### 2.5. Statistical analysis

In the current study, we applied the sample weights, stratifications, and clustering in the NHANES dataset in all statistical analyses to consider the multistage and complex sampling design employed to ensure the representation of a noninstitutionalized civilian U.S. population. Continuous variables were represented as weighted mean and standard error, while categorical variables were set as weighted proportions and 95% confidence interval (CI). To assess baseline characteristics across the 4 groups categorized by GNRI quartiles, survey-weighted linear regression was utilized for continuous variables, while a survey-weighted chi-square test was employed for categorical variables. Multivariable logistic regression analysis was used to evaluate the connection between GNRI and UI. In model 1, no covariates were adjusted. In model 2, gender, age, and race were adjusted. In model 3, gender, age, race, education level, marital status, the family PIR, BMI, smoking status, alcohol intake, vigorous activity, moderate activity, diabetes, and hypertension were adjusted. The use of smooth curve fitting and generalized additive models allowed for an examination of the potential segmentation of GNRI into different intervals, thereby enabling an assessment of the nonlinear relationship between the GNRI and UI. Subgroup analysis was employed to explore the stability of the association between GNRI and UI across various stratifications. Statistical significance was established at a 2-sided *P* value < .05. EmpowerStats (http://www.empowerstats.com, X&Y Solutions, Inc.) and statistical software packages R (http://www.R-project.org; The R Foundation) were used in all statistical analyses.

## 3. Results

### 3.1. Participant characteristics

The current study included a sample of 11,324 participants with an average age (standard error) of 70.51 (0.10) years. The weighted proportions of male and female participants were 45.0% and 55.0%, respectively. Of the study individuals, 32.1% disclosed a history of SUI, 36.5% manifested a previous occurrence of UUI, and 17.8% reported symptoms indicative of MUI. Based on the quartiles of GNRI, participants in the highest quartile had a lower risk of 3 types of UI (all *P* < .05). More detailed baseline characteristics are presented in Table 1.

**Table 1:** Baseline characteristics of participants by the GNRI quartiles, weighted.

	Total	Q1	Q2	Q3	Q4	*P* value
Age (yr), mean (SE)	70.51 (0.10)	71.66 (0.22)	70.81 (0.17)	70.42 (0.22)	69.80 (0.14)	< .001
BMI (kg/m^2^), mean (SE)	29.00 (0.09)	29.14 (0.23)	29.83 (0.14)	28.53 (0.20)	28.44 (0.12)	< .001
Gender, % (95% CI)						< .001
Male	45.0 (44.0–45.9)	34.6 (31.5–37.8)	41.4 (39.3–43.6)	45.6 (42.2–49.1)	52.1 (50.3–53.8)	
Female	55.0 (54.1–56.0)	65.4 (62.2–68.5)	58.6 (56.4–60.7)	54.4 (50.9–57.8)	47.9 (46.2–49.7)	
Age, % (95% CI)						< .001
< 70	49.8 (48.4–51.2)	43.0 (40.1–45.9)	47.7 (45.2–50.2)	50.9 (47.6–54.2)	54.1 (52.0–56.2)	
≥ 70	50.2 (48.8–51.6)	57.0 (54.1–59.9)	52.3 (49.8–54.8)	49.1 (45.8–52.5)	45.9 (43.8–48.0)	
Race/Ethnicity, % (95% CI)						< .001
Mexican American	3.8 (3.0–4.9)	4.4 (3.3–5.7)	3.7 (2.8–4.7)	3.9 (2.8–5.2)	3.8 (2.9–4.9)	
Other Hispanic	3.2 (2.6–4.0)	3.0 (2.3–4.1)	3.5 (2.8–4.4)	3.3 (2.4–4.4)	3.1 (2.4–4.0)	
Non-Hispanic White	80.2 (78.1–82.1)	74.4 (71.4–77.2)	80.0 (77.7–82.1)	80.7 (77.7–83.3)	82.6 (80.3–84.8)	
Non-Hispanic Black	7.9 (6.8–9.1)	13.2 (11.3–15.4)	8.4 (7.1–9.9)	7.4 (6.1–9.0)	5.3 (4.5–6.3)	
Other race	4.9 (4.2–5.6)	5.0 (3.8–6.6)	4.4 (3.7–5.4)	4.8 (3.7–6.1)	5.2 (4.2–6.4)	
Education level, % (95% CI)						< .001
< high school	19.8 (18.3–21.4)	24.1 (21.7–26.6)	20.3 (18.4–22.4)	19.4 (16.9–22.2)	17.7 (15.8–19.7)	
High school or GED	25.8 (24.6–27.1)	27.4 (24.8–30.1)	26.8 (24.4–29.3)	21.8 (18.7–25.2)	25.9 (23.9–28.0)	
> high school	54.4 (52.6–56.2)	48.6 (45.5–51.6)	52.9 (50.1–55.7)	58.8 (54.8–62.7)	56.5 (53.6–59.3)	
Marital status, % (95% CI)						< .001
Living alone	36.6 (35.2–38.1)	46.4 (43.7–49.2)	38.7 (36.6–40.9)	32.8 (29.4–36.2)	32.1 (30.2–34.2)	
Married or living with partner	63.4 (61.9–64.9)	53.6 (50.9–56.3)	61.3 (59.1–63.4)	67.3 (63.8–70.6)	67.9 (65.8–69.8)	
Family PIR, % (95% CI)						< .001
< 1.3	16.4 (15.2–17.8)	21.1 (18.8–23.6)	17.1 (15.5–18.8)	16.2 (13.9–18.8)	14.0 (12.5–15.7)	
> 1.3 and ≤ 3.5	38.6 (37.0–40.2)	39.0 (35.6–42.6)	38.7 (36.4–41.0)	37.5 (33.9–41.3)	38.6 (36.3–41.1)	
> 3.5	37.1 (34.9–39.3)	30.7 (26.5–35.3)	36.4 (34.0–38.9)	37.4 (33.1–41.9)	40.2 (37.5–42.9)	
Unclear	8.0 (7.1–8.8)	9.2 (7.2–11.6)	7.9 (6.7–9.2)	8.9 (7.0–11.3)	7.1 (6.1–8.4)	
BMI, % (95% CI)						< .001
< 25	25.7 (24.5–27.0)	33.9 (30.9–37.0)	20.9 (19.1–22.8)	29.7 (26.5–33.1)	24.8 (22.9–26.8)	
≥ 25 and < 30	37.0 (35.7–38.2)	24.0 (21.2–27.0)	36.4 (34.2–38.7)	34.8 (31.5–38.2)	43.8 (41.8–45.8)	
≥ 30	37.3 (36.0–38.7)	42.1 (39.1–45.2)	42.7 (40.5–44.9)	35.5 (31.8–39.3)	31.5 (29.6–33.4)	
Hypertension, % (95% CI)						.225
No	30.8 (29.4–32.3)	28.8 (25.8–31.9)	30.1 (27.7–32.6)	33.0 (29.7–36.5)	31.5 (29.5–33.6)	
Yes	69.2 (67.7–70.6)	71.3 (68.1–74.2)	69.9 (67.4–72.3)	67.0 (63.5–70.3)	68.5 (66.4–70.5)	
Diabetes, % (95% CI)						< .001
No	77.3 (76.3–78.3)	73.2 (70.3–76.0)	76.3 (74.6–77.9)	79.5 (76.7–82.0)	79.1 (77.2–80.78)	
Yes	22.7 (21.7–23.7)	26.8 (24.0–29.7)	23.7 (22.1–25.5)	20.5 (18.0–23.3)	21.0 (19.2–22.8)	
Vigorous activity, % (95% CI)						< .001
No	85.1 (83.9–86.3)	90.1 (87.8–92.1)	85.1 (83.1–86.9)	85.0 (82.2–87.5)	83.0 (81.2–84.8)	
Yes	14.9 (13.7–16.1)	9.9 (8.0–12.2)	14.9 (13.1–16.9)	15.0 (12.5–17.8)	17.0 (15.3–18.8)	
Moderate activity, % (95% CI)						< .001
No	59.5 (57.8–61.2)	68.4 (65.0–71.6)	59.9 (57.2–62.5)	59.7 (56.4–62.9)	55.4 (52.7–58.1)	
Yes	40.5 (38.8–42.2)	31.6 (28.4–35.0)	40.2 (37.6–42.8)	40.4 (37.1–43.7)	44.6 (41.9–47.3)	
Smoking status, % (95% CI)						< .001
Current smokers	11.0 (10.3–11.9)	15.4 (13.1–17.9)	10.9 (9.7–12.3)	10.9 (8.9–13.4)	9.4 (8.2–10.7)	
Former smokers	40.0 (38.7–41.4)	37.8 (35.0–40.6)	38.4 (36.1–40.7)	38.5 (35.1–41.9)	42.9 (40.7–45.0)	
Nonsmokers	49.0 (47.5–50.4)	46.9 (43.9–49.8)	50.7 (48.2–53.2)	50.6 (46.8–54.5)	47.8 (45.4–50.2)	
Alcohol intaking, % (95% CI)						< .001
Nondrinkers	27.7 (26.0–29.6)	28.4 (25.5–31.4)	28.3 (26.2–30.5)	29.9 (26.8–33.3)	26.2 (23.8–28.7)	
Drinkers	55.6 (53.3–57.9)	42.1 (38.4–45.9)	50.4 (47.7–53.0)	57.1 (53.1–61.1)	65.3 (62.3–68.1)	
Unclear	16.6 (14.7–18.7)	29.5 (25.4–34.1)	21.4 (18.9–24.1)	13.0 (10.0–16.7)	8.6 (6.6–11.1)	
SUI, % (95% CI)						< .001
No	67.8 (66.7–69.0)	60.5 (57.3–63.6)	66.1 (63.8–68.3)	69.4 (66.3–72.4)	71.8 (69.9–73.6)	
Yes	32.2 (31.0–33.3)	39.5 (36.4–42.7)	33.9 (31.7–36.2)	30.6 (27.6–33.7)	28.2 (26.4–30.1)	
MUI, % (95% CI)						< .001
No	63.5 (62.1–64.8)	55.2 (52.5–58.0)	60.5 (58.2–62.8)	64.8 (61.4–68.1)	68.9 (66.5–71.2)	
Yes	36.5 (35.2–38.0)	44.8 (42.0–47.5)	39.5 (37.3–41.8)	35.2 (31.9–38.6)	31.1 (28.8–33.5)	
UUI, % (95% CI)						< .001
No	82.2 (81.2–83.2)	75.6 (72.8–78.3)	80.2 (78.5–81.9)	82.9 (80.2–85.4)	86.4 (84.7–88.0)	
Yes	17.8 (16.8–18.8)	24.4 (21.7–27.2)	19.8 (18.1–21.5)	17.1 (14.7–19.8)	13.6 (12.0–15.3)	

Q1–Q4: quartile 1 to quartile 4.

BMI = body mass index, CI = confidence interval, GED = general educational development, GNRI = geriatric nutritional risk index, MUI = mixed urinary incontinence, PIR = poverty-to-income ratio, SE = standard error, SUI = stress urinary incontinence, UUI = urgency urinary incontinence.

### 3.2. The association between GNRI and UI

**Figure 2.**
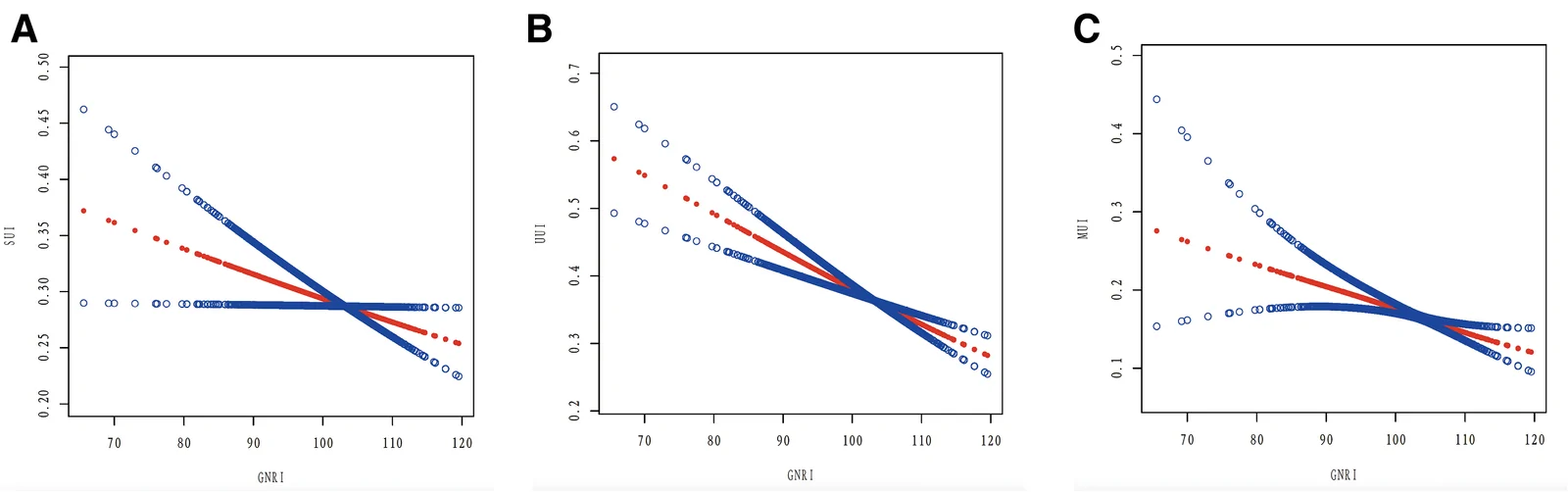
Smooth curve fitting for the relationship between GNRI and 3 types of UI. (A–C) represent the linear connections between GNRI and SUI, UUI, and MUI, respectively. The area between 2 blue dotted lines represents a 95% CI. The red dotted line suggests the negative linear relationship between GNRI and 3 types of UI. CI = confidence interval, GNRI = geriatric nutritional risk index, MUI = mixed urinary incontinence, SUI = stress urinary incontinence, UI = urinary incontinence, UUI = urgency urinary incontinence.

Multivariable logistic regression was used to assess the relationship between GNRI and UI in crude (model 1), minimally adjusted (model 2), and fully adjusted (model 3) models. The results demonstrated that the GNRI was negatively related to the likelihood of UI. In model 3, there was a decrease in the risk of UI with each successive unit increase in GNRI (SUI: odds ratio [OR] = 0.986, 95% CI: 0.974–0.999; UUI: OR = 0.972, 95% CI: 0.961–0.983; MUI: OR = 0.972, 95% CI: 0.957–0.987, all *P* < .05). After converting GNRI into a categorical variable according to the GNRI quartiles (Q1–Q4), the negative connection still existed. In model 3, survey individuals belonging to the highest GNRI quartile (Q4) had a lower likelihood of UI compared to participants in the lowest group (Q1) (SUI: OR = 0.797, 95% CI: 0.672–0.945; UUI: OR = 0.722, 95% CI: 0.615–0.848; MUI: OR = 0.674, 95% CI: 0.538–0.845, all *P* for trend < .05). More information is presented in Table 2. Furthermore, the smooth curve fitting revealed a negative linear association between GNRI and 3 types of UI (Fig. 2).

**Table 2:** Association between GNRI and urinary incontinence, weighted.

SUI	OR (95% CI), *P* value		
	Model 1	Model 2	Model 3
Continuous*	0.964 (0.953–0.976), < .001	0.981 (0.968–0.993), .004	0.986 (0.974–0.999), .031
Categories†			
Q1	Reference	Reference	Reference
Q2	0.785 (0.672–0.918), .003	0.835 (0.708–0.986), .036	0.832 (0.704–0.983), .033
Q3	0.674 (0.551–0.825), < .001	0.756 (0.609–0.939), .013	0.791 (0.637–0.980), .035
Q4	0.601 (0.507–0.712), < .001	0.754 (0.634–0.897), .002	0.797 (0.672–0.945), .010
*P* for trend	< .001	.004	.034

UUI	OR (95% CI), *P* value		
	Model 1	Model 2	Model 3
Continuous*	0.953 (0.943–0.964), < .001	0.964 (0.954–0.975), < .001	0.972 (0.961–0.983), < .001
Categories†			
Q1	Reference	Reference	Reference
Q2	0.806 (0.700–0.928), .003	0.860 (0.739–1.000), .050	0.869 (0.742–1.017), .084
Q3	0.670 (0.565–0.794), < .001	0.742 (0.617–0.893), .002	0.805 (0.674–0.961), .018
Q4	0.557 (0.481–0.645), < .001	0.654 (0.558–0.767), < .001	0.722 (0.615–0.848), < .001
*P* for trend	< .001	< .001	< .001

MUI	OR (95% CI), *P* value		
	Model 1	Model 2	Model 3
Continuous*	0.949 (0.936–0.963), < .001	0.963 (0.948–0.978), < .001	0.972 (0.957–0.987), < .001
Categories†			
Q1	Reference	Reference	Reference
Q2	0.765 (0.637–0.919), .005	0.822 (0.673–1.000), .057	0.840 (0.686–1.030), .093
Q3	0.640 (0.515–0.795), < .001	0.726 (0.577–0.913), .007	0.799 (0.638–1.002), .052
Q4	0.487 (0.394–0.602), < .001	0.601 (0.482–0.750), < .001	0.674 (0.538–0.845), < .001
*P* for trend	< .001	< .001	< .001

Model 1: Unadjusted.

Model 2: Adjusted for gender, age, and race/ethnicity.

Model 3: Adjusted for gender, age, race/ethnicity, education level, marital status, the family PIR, BMI, smoking status, alcohol intaking, vigorous activity, moderate activity, diabetes, and hypertension.

Q1–Q4: quartile 1 to quartile 4.

BMI = body mass index, CI = confidence interval, GNRI = geriatric nutritional risk index, MUI = mixed urinary incontinence, OR = odds ratio, PIR = poverty-to-income ratio, SUI = stress urinary incontinence, UUI = urgency urinary incontinence.

* “Continuous” means that GNRI is designed as a continuous variable.

† “Categories” represents that GNRI is set as a categorical variable and divided into 4 groups (quartile 1-quartile 4).

### 3.3. Subgroup analysis

**Figure 3.**
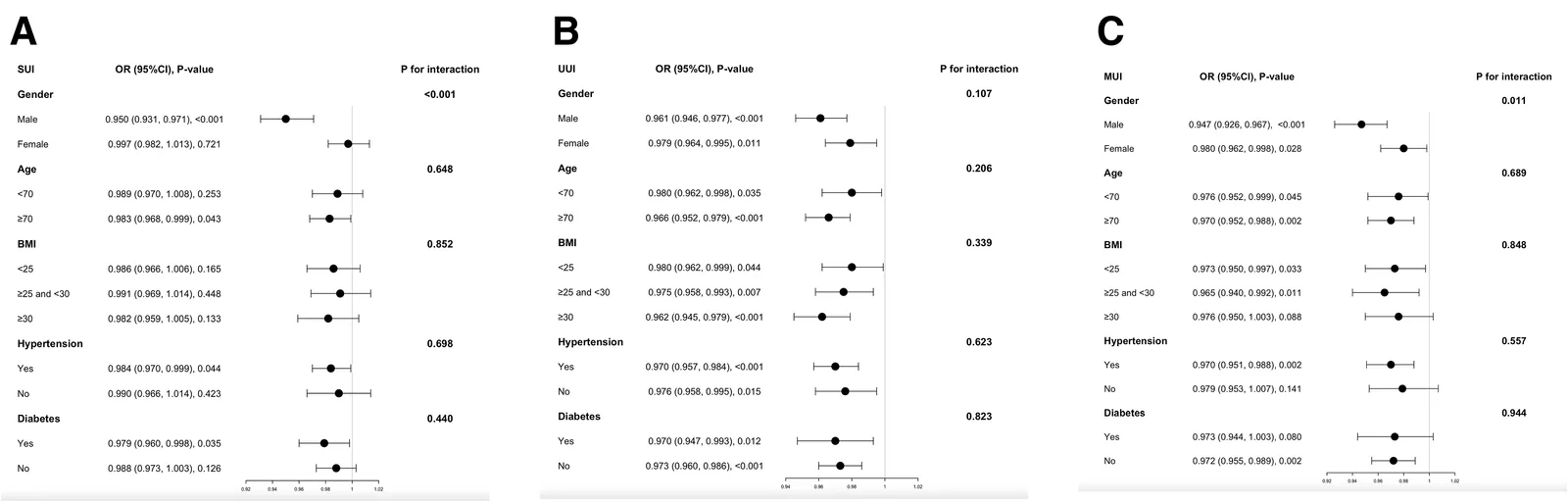
Subgroup analysis for the association between GNRI and 3 types of UI. (A–C) present the subgroup analysis results of the associations between the GNRI index and SUI, UUI, and MUI, respectively, across different stratifications. All stratified factors include gender, age, race/ethnicity, education level, marital status, the family PIR, smoking status, alcohol intake, vigorous activity, moderate activity, diabetes, and hypertension, except the stratified factor itself. BMI = body mass index, CI = confidence interval, GNRI = geriatric nutritional risk index, MUI = mixed urinary incontinence, OR = odds ratio, PIR = poverty-to-income ratio, SUI = stress urinary incontinence, UI = urinary incontinence, UUI = urgency urinary incontinence.

Subgroup analysis stratified by gender, age, BMI, hypertension, and diabetes was conducted to explore the stability of the negative relationship between GNRI and UI. In Figure 3A, the negative association between GNRI and SUI was stronger in males compared to females. Additionally, a difference was observed in the relationship between GNRI and MUI as shown in Figure 3C (*P* for interaction < .05). In UUI, there were no significant differences in subgroups (Fig. 3B). Therefore, gender may be a potential factor influencing the connection. Moreover, tests for interaction were not significant in other stratifications. More detailed data are shown in Figure 3.

## 4. Discussion

To our knowledge, this is the first study to examine the link between nutritional status and the occurrence of UI in older adults, based on a large-scale demographic survey of the NHANES database in the U.S. The results of this study show that an increase in GNRI in older adults is associated with a reduced risk of UI after adjusting for covariates, regardless of the type of UI. The smooth curve fitting shows a negative linear relationship between GNRI and UI. Moreover, the subgroup analysis demonstrates a stronger association between GNRI and both SUI and MUI in males than in females. This gender difference warrants deeper reflection.

UI causes distress in the lives of many older people, especially older women. There is no consensus on the risk factors for UI. Current evidence supports that older age, obesity, and post-hysterectomy are important risk factors for UI.^[18–22]^ Some chronic diseases may also be contributing factors to incontinence, such as stroke, diabetes, and hypertension.^[5,23]^ However, the evidence on the relationship between nutritional status and the prevalence of UI among older adults remains insufficient.

The GNRI is a composite indicator of nutritional status that integrates serum albumin levels and BMI, thereby minimizing the confounding effect between them. Several studies have previously explored the relationship between serum albumin and UI. Xu et al reported that elevated serum albumin levels are beneficial in reducing the risk of SUI in women based on a large population-based study.^[9]^ For each 1 g/dL increase in serum albumin, the risk of moderate/severe SUI was reduced by 23.7%. In addition, in a study on overactive bladder syndrome, researchers observed that serum albumin levels tended to be lower in patients with nocturia and those with UUI.^[24]^ These findings suggest that serum albumin levels may reflect the prevalence of UI to some extent, and UI may be linked to nutritional status. The evidence for BMI and obesity as risk factors for UI appears to be stronger. Several studies have demonstrated that BMI can be an independent predictor of UI, especially for female SUI.^[21,25–28]^ Clinical trials have confirmed the therapeutic effect of bariatric surgery for UI.^[26,29]^ A systematic review and meta-analysis suggest that weight loss may be considered a standard treatment for UI.^[30]^ Moderate BMI levels are also an important indicator for assessing nutritional status in older adults. In this study, the GNRI combined patients’ serum albumin levels and BMI to comprehensively assess their nutritional status. Therefore, GNRI may be a predictor of UI development. While the current study primarily focuses on the influence of poor nutritional status on the prevalence and severity of UI among older adults, it is important to acknowledge the potential for a bidirectional relationship. A cross-sectional study in Taiwan revealed that UI was reported by approximately one-fifth of the participants and was significantly connected to more comorbidity, poorer physical function, and worse nutritional status.^[31]^ Rose et al showed that in nursing homes, greater severity of incontinence was strongly associated with poorer nutritional status, increased dementia-related symptoms, and impaired mobility.^[32]^ The potential impact of UI on nutritional status merits further investigation. Several possible mechanisms could underline this reverse relationship. Older people experiencing UI may restrict their fluid and food intake for managing or avoiding incontinent episodes, which can result in dehydration and insufficient nutrient intake. Moreover, UI is often associated with psychosocial consequences such as embarrassment, social withdrawal, and depressive symptoms, all of which can negatively impact appetite and eating behavior. The potential bidirectional relationship between GNRI and UI may affect the interpretation of our findings. While poor nutritional status may increase the risk of UI, UI itself could contribute to nutritional decline through factors such as reduced appetite or intentional fluid and food restriction. Recognizing this complexity highlights the need to pay attention to both nutrition and urinary health in older adults.

It is unclear why nutritional status in older adults affects the development of UI, and there may be several underlying mechanisms. Pelvic floor muscle hypofunction is a possible pathologic mechanism for the development of UI.^[33,34]^ Several studies have shown a correlation between nutritional status and muscle strength.^[35–37]^ Better nutritional status in older adults may affect the strength and function of the pelvic floor muscles and may thereby reduce the risk of UI. Estrogen levels are also an important factor in the progression of UI.^[38,39]^ Nutritional status has also been associated with hormone levels.^[40,41]^ The effect of nutritional status on hormones may also be one of the potential mechanisms for their association with the development of UI. In this study, the subgroup analysis demonstrates that a stronger association between GNRI and UI is observed in male participants compared to females. This sex-specific difference warrants further discussion. There are several possible explanations for this kind of difference. First, malnutrition was related to an increased risk of sarcopenia.^[42]^ Men normally have greater baseline muscle mass and larger pelvic floor muscles. Malnutrition-induced sarcopenia may lead to a more obvious loss of urethral support and detrusor contractility in men. Indeed, Langston et al showed that higher limb muscle strength was associated with milder lower urinary tract symptoms in older men, suggesting that sarcopenia exacerbated male UI.^[43]^ Moreover, a large population survey found that female UI is strongly associated with obesity, physical inactivity, and smoking, whereas male UI is driven by age, frailty, and comorbid diseases.^[44]^ Therefore, a poorer nutritional status would have a larger effect on UI risk in males.

Our study utilized a substantial dataset derived from the NHANES database and accounted for the sampling design and weighting to accurately describe the general population of the U.S. Nevertheless, several limitations are inherent in the present investigation. First, the cross-sectional design of this study limits the exploration of causal association between GNRI and UI but also fails to rule out the potential impact of reverse causation. Specifically, it remains unclear whether poor nutritional status (reflected by low GNRI scores) contributes to the development or progression of UI, or if UI itself leads to reduced dietary intake, weight loss, and subsequent declines in nutritional status; this bidirectional relationship cannot be disentangled with the current cross-sectional data. In addition, the NHANES database reflects only the U.S. population, limiting the generalizability of the findings to other countries. Therefore, the association between GNRI and UI warrants validation through further studies across diverse national populations. Finally, while we adjusted for several potential covariates in the present study, residual confounding remains an unavoidable limitation. It is difficult to eliminate the impact of other conceivable confounding factors. In addition to commonly controlled sociodemographic and clinical factors, several other covariates may influence the relationship between GNRI and UI, such as pelvic floor muscle strength, caregiver support, and hormonal levels. However, due to the limitation of NHANES database, we could not include these covariates in the current study. It is recommended that future studies include these factors to enhance the robustness and generalizability of the present results.

## 5. Conclusions

To the best of our knowledge, this represents the first cross-sectional investigation delving into the association between GNRI and UI within the old adults (> 60 years old) of the U.S. Our study indicated a negative relationship between GNRI and all 3 types of UI (SUI, UUI, and MUI). Notably, even upon stratification of GNRI into quartiles, the negative connection remained stable. The relation between GNRI and SUI, MUI was stronger in men than in women. Nevertheless, further research is necessary to corroborate and extend our findings.

## Acknowledgments

All authors express their gratitude to all participants and personnel involved in the NHANES.

## Author contributions

**Conceptualization:** Juan Xu, Xiang Li, Shu Wen.

**Data curation:** Juan Xu, Shangqi Cao, Xu Hu.

**Formal analysis:** Juan Xu, Shangqi Cao.

**Funding acquisition:** Shu Wen.

**Methodology:** Juan Xu, Shangqi Cao, Xu Hu.

**Resources:** Shangqi Cao.

**Software:** Juan Xu, Xu Hu.

**Supervision:** Xiang Li, Shu Wen.

**Writing – original draft:** Juan Xu, Shangqi Cao, Xu Hu.

**Writing – review & editing:** Juan Xu, Xiang Li, Shu Wen.
